# Laparoscopy endoscopy cooperative surgery for gastric plexiform fibromyxoma: a case report

**DOI:** 10.1186/s40792-016-0249-z

**Published:** 2016-10-29

**Authors:** Yoshikage Inoue, Shutaro Gunji, Kazutaka Obama, Hiroshi Okabe, Yoshiharu Sakai

**Affiliations:** 1 Department of Surgery, Kyoto University Graduate School of Medicine, Yoshida Konoecho, Sakyo-ku, Kyoto-shi, Kyoto Japan; 2 Department of Surgery, Otsu Municipal Hospital, 2-9-9, Motomiya, Otsu-shi, Shiga Japan

## Abstract

**Background:**

Gastric submucosal tumors are commonly treated by partial resection under laparoscopy. However, the surgical resection of gastric submucosal tumors sometimes causes deformation of the stomach, especially in the case of intraluminal tumors located near the pylorus or esophagogastric junction. Such deformations can result in impaired diet intake and reduced quality of life. Laparoscopic endoscopic cooperative surgery has been developed to overcome these problems. This is the first report to describe a case of gastric plexiform fibromyxoma, a rare gastric submucosal tumor, that was successfully resected by laparoscopic endoscopic cooperative surgery.

**Case presentation:**

A 36-year-old Japanese woman presented with epigastric pain and anemia. Gastrointestinal endoscopy revealed a submucosal tumor in the gastric antrum. Because a definitive diagnosis could not be obtained and the tumor was located near the pylorus, we performed laparoscopic endoscopic cooperative surgery as diagnostic therapy. The postoperative course was favorable with no complications, such as delayed gastric emptying or outlet obstruction. The tumor was pathologically diagnosed as gastric plexiform fibromyxoma.

**Conclusions:**

Laparoscopic endoscopic cooperative surgery is a useful approach for diagnostic therapy for rare submucosal tumors to avoid the deformation of the stomach, especially when the tumor is located near the pylorus.

## Background

Laparoscopic endoscopic cooperative surgery (LECS) was first introduced by Hiki et al. [1] to determine the appropriate resection line for gastric submucosal tumors (SMTs) by applying the endoscopic submucosal dissection (ESD) technique. An appropriate resection line with a minimal surgical margin can reduce the size of the defect in the gastric wall, which can prevent deformation of the stomach. Subsequently, LECS has mainly been applied to the resection of gastrointestinal stromal tumors (GISTs), because they do not require large surgical margins.

The great majority of gastric mesenchymal tumors are GISTs, which present with immunopositivity for CD34 or c-kit (CD117) and mutations in the *KIT* gene. Although advances in molecular pathology have made definitive diagnoses easier to obtain [2–4], in the case of tumors other than GISTs, some tumors remain difficult to diagnose because of their rarity and the possibility of a wide range of differential diagnoses. However, an accurate diagnosis is essential since the clinical course or prognosis differs among various tumors [5].

The tumor in this report was diagnosed as a plexiform fibromyxoma, which is a rare type of mesenchymal tumor. It is a benign tumor that has recently been defined as a multinodular myxoid tumor involving the gastric antrum with a peculiar plexiform growth pattern, myxoid stroma, prominent vasculature, and spindle cells with myofibroblastic differentiation [6, 7]. Since Takahashi et al. first described this tumor in 2007, only 25 cases have been reported in the literature [5–13].

Here, we present a case of a rare gastric submucosal tumor, plexiform fibromyxoma, that was successfully resected by LECS. We also discuss the clinical advantages of LECS in resecting gastric SMTs that are located in the vicinity of the pylorus as well as the characteristics of plexiform fibromyxoma.

## Case presentation

**Fig. 1:**
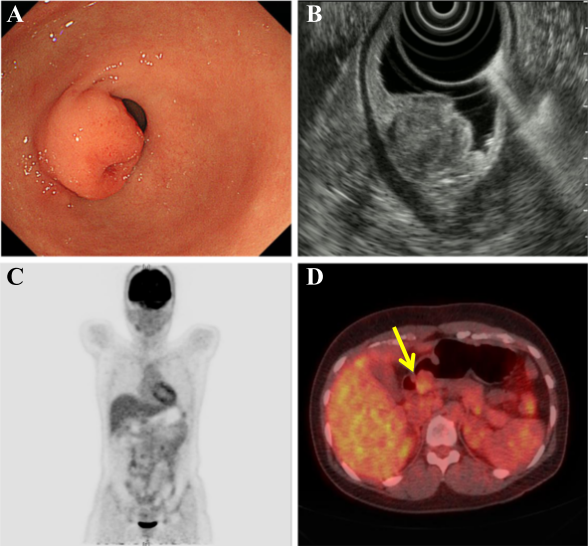
**a** Upper gastrointestinal endoscopy revealed an elevated mucosal lesion in the pyloro-antral region with erosive changes. **b** Endoscopic ultrasonography revealed a heterogeneous tumor measuring 20 mm that was not connected to the muscularis propria of the stomach. **c**, **d** A positron emission tomography/computed tomography scan did not show significant uptake of ^18^F-fluorodeoxyglucose into the tumor (*arrow*)

A 36-year-old woman presented with epigastric pain and anemia. Gastrointestinal endoscopy revealed an elevated mucosal lesion with linear ulceration in the anterior wall of the gastric antrum, located very close to the pylorus (Fig. 1a). A definitive diagnosis could not be obtained, even with an incisional biopsy. She was referred to our hospital for further investigation and treatment.

The patient’s medical history and family history were noncontributory. Laboratory evaluation, including tumor marker levels, showed results within the normal range except for the presence of anemia. Endoscopic ultrasonography showed a heterogeneous echoic tumor in the submucosal layer not involving the muscularis propria (Fig. 1b). Computed tomography (CT) showed a nodular soft tissue mass in the gastric antrum, with no apparent metastatic lesions. There was no significant uptake of ^18^F-fluorodeoxyglucose into the tumor on a positron emission tomography/CT scan (Fig. 1c, d).

**Fig. 2:**
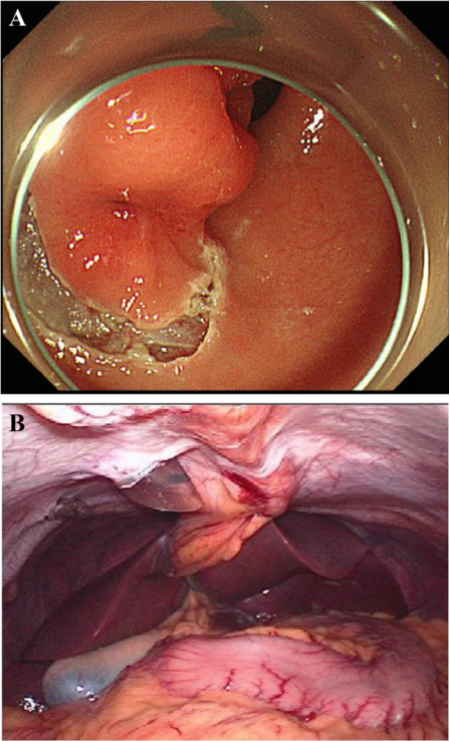
**a** Circumferential marking of the tumor using the endoscopic submucosal dissection technique with an insulation-tipped knife. **b** The tumor did not show any changes on the gastric wall

The results were inconclusive for excluding malignant potential of the tumor. Additionally, resection with ESD technique was difficult to perform, given the high risk of perforation during the procedure. Under the presumption that the tumor was a GIST, schwannoma, or other types of submucosal tumors, we sought to achieve a definitive diagnosis by radical resection for total biopsy with minimal margins. Among the various surgical procedures, we chose LECS, with more than usual caution on the prevention of gastric juice leakage into the intraperitoneal cavity (Fig. 2a, b). LECS was performed as described by Hiki et al. [1]. Briefly, it is executed in three main parts. First, the endoscopist marks the smallest negative margin around the tumor using the ESD technique. Second, the submucosal incision is extended toward the serosa passing through the muscularis propria, resulting in a small artificial perforation. Finally, a surgeon resects the tumor laparoscopically with the guidance of the endoscope and surgical marks. The defect in the gastric wall is then closed either with the continuous hand-sewn technique or using linear staples. We chose the continuous hand-sewn technique, because a portion of the suture line was on the pylorus.

**Fig. 3:**
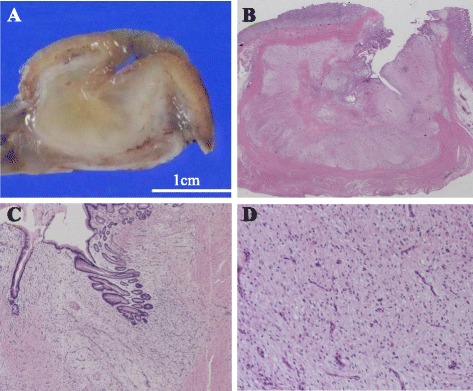
**a** Gross appearance of the tumor showed a submucosal tumor with ulceration. The cross-sectional examination showed a gray white tumor with a clear boundary. **b** Hematoxylin and eosin staining showed lobulated or fused multinodular growth. **c** Low-power field: the tumor was located in the lamina propria mucosae and protruded into the muscularis propria. **d** High-power field: bland spindle cell proliferation with myxoid stroma and prominent vasculature was noted

The resected specimen appeared as a gray white solid tumor, measuring 25 × 22 × 20 mm (Fig. 3a, b), with negative margins. The tumor was located in the lamina propria mucosae and protruded into the muscularis propria, showing no necrosis or lymphovascular invasion. Histopathological examination revealed partial dense collagenous matrices and networks of fine capillary-caliber blood vessels, showing infiltration of lymphocytes, plasma cells, and mast cells. The tumor demonstrated lobular or fused nodular growth of spindle cells without atypical cytology, with abundant alcian blue-positive myxoid extracellular matrix (Fig. 3c, d).

**Fig. 4:**
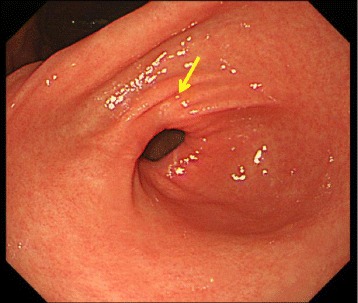
Follow-up endoscopy showed no significant gastric deformity, with minimal involvement of scar tissues on the pylorus (*arrow*)

After the confirmation of the diagnosis and the decision that additional treatments were unnecessary, the patient was discharged on postoperative day 8 with no complications, such as delayed gastric emptying or outlet obstruction. She has been followed in the outpatient clinic and shown no signs of recurrence or metastasis. No distortion of the stomach was evident on endoscopy (Fig. 4).

## Conclusions

Gastric plexiform fibromyxomas are usually resected by partial gastrectomy or distal gastrectomy, except for a rare sessile polypoid case reported by Kang et al. [8, 9]. As in the case of GISTs, this rare, benign tumor can be resected with minimal surgical margins since no cases of recurrence or metastasis have been reported to date. However, the indication of resection for this tumor remains unclear because of its benign character and rarity. In the present case, because we could not make a definitive diagnosis of this gastric SMT before surgery, the most appropriate option was radical resection of the tumor for total biopsy to exclude the malignant potential of this SMT.

A major issue associated with surgery for gastric SMTs is the difficulty in determining an appropriate dissection line, especially for small intraluminal tumors. In our case, because the tumor was intraluminal and measured only 2 cm in diameter, we were unable to detect the location of the tumor using laparoscopy alone. Dissection without accurate detection of the tumor extent may result in excessive resection of the gastric wall, leading to deformation of the stomach and impaired gastric function. In particular, such effects are exacerbated in the case of tumors located near the pylorus or esophagogastric junction. Among the operative procedures developed to overcome these problems, LECS confers the advantage of direct observation of the tumor and, consequently, the precise determination of surgical margins. In recent years, its applications have broadened not only to gastric submucosal tumors but also to early gastric carcinomas that are difficult to resect with ESD [14].

Takahashi et al. first reported two cases showing a plexiform angiomyxoid myofibroblastic tumor in 2007 [7]. Miettinen et al. proposed the alternative name of plexiform fibromyxoma in 2009 [6], which was accepted by the WHO Classification of Tumours of the Digestive System. Plexiform fibromyxoma and plexiform angiomyxoid myofibroblastic tumor are closely related entities showing variable fibroblastic-myofibroblastic differentiation [6, 10, 11]. The clinical presentation is generally similar to that of GISTs, with gastrointestinal bleeding being the most common symptom. In some cases, pyloric obstruction with weight loss may be noted. Miettinen et al. reported that they found 12 cases of this tumor among over 4200 mesenchymal tumors at various gastrointestinal sites, and all of them were located in the gastric antrum. They assumed that this fact may be related to an anatomically restricted progenitor cell population [6].

In the present case, tumor resection for total biopsy using LECS led to the correct diagnosis without impairing the patient’s quality of life. With the correct diagnosis, we were able to choose the appropriate treatment, avoiding unnecessary therapy. Our results suggest that LECS is a useful procedure for achieving a definitive diagnosis for rare submucosal tumors such as plexiform fibromyxoma, which cannot be easily diagnosed before surgery.

## Abbreviations

CT: Computed tomography
ESD: Endoscopic submucosal dissection
FDG: ^18^F-fluorodeoxyglucose
GIST: Gastrointestinal stromal tumors
LECS: Laparoscopy endoscopy cooperative surgery
PET: Positron emission tomography
SMT: Submucosal tumor
